# In Vitro and In Vivo Antioxidant Properties of *Taraxacum officinale* in *N*ω-Nitro-l-Arginine Methyl Ester (L-NAME)-Induced Hypertensive Rats

**DOI:** 10.3390/antiox8080309

**Published:** 2019-08-15

**Authors:** Olukayode O. Aremu, Adebola O. Oyedeji, Opeoluwa O. Oyedeji, Benedicta N. Nkeh-Chungag, Constance R. Sewani Rusike

**Affiliations:** 1 Department of Human Biology, Faculty of Health Sciences, Walter Sisulu University, Mthatha 5117, South Africa; 2 Department of Chemistry, Faculty of Sciences, Walter Sisulu University, Mthatha 5117, South Africa; 3 Department of Chemistry, Faculty of Sciences, University of Fort Hare, Alice 5700, South Africa; 4 Department of Biological and Environmental Sciences, Faculty of Sciences, Walter Sisulu University, Mthatha 5117, South Africa

**Keywords:** oxidative stress, acute toxicity, antioxidants, L-NAME, DPPH, FRAP, ABTS

## Abstract

Oxidative stress has gained attention as one of the fundamental mechanisms responsible for the development of hypertension. The present study investigated in vitro and in vivo antioxidant effects of 70% ethanol-water (*v*/*v*) leaf and root extracts of *T. officinale* (TOL and TOR, respectively). Total phenolic and flavonoid content of plant extracts were assessed using Folin Ciocalteau and aluminium chloride colorimetric methods; while, 2,2-diphenyl-1-picrlhydrazyl (DPPH), 2,2-azinobis (3-ethylbenzothiazoline-6-sulfonic acid (ABTS) and ferric reducing antioxidant power (FRAP) protocols were used to determine the free radical scavenging and total antioxidant capacities (TAC), respectively. The in vivo total antioxidant capacity and malondialdehyde acid (MDA) levels for lipid peroxidation tests were performed on organ homogenate samples from *N*ω-nitro-L-arginine methyl ester (L-NAME)-induced hypertensive rats treated with leaf extract, TOL (500 mg/kg/day) and TOR (500 mg/kg/day) for 21 days. Results showed that compared to TOR, TOL possessed significantly higher (*p* < 0.01) polyphenol (4.35 ± 0.15 compared to 1.14 ± 0.01) and flavonoid (23.17 ± 0.14 compared to 3 ± 0.05) content; free radical scavenging activity (EC_50_ 0.37 compared to 1.34 mg/mL) and total antioxidant capacities (82.56% compared to 61.54% ABTS, and 156 ± 5.28 compared to 40 ± 0.31 FRAP) and both extracts showed no toxicity (LD_50_ > 5000 mg/kg). TOL and TOR significantly (*p* < 0.01) elevated TAC and reduced MDA levels in targets organs. In conclusion, *T. officinale* leaf extract possesses significant anti-oxidant effects which conferred significant in vivo antioxidant protection against free radical-mediated oxidative stress in L-NAME-induced hypertensive rats.

## 1. Introduction

Reactive oxygen species (ROS)-mediated oxidative stress is the most influencing pathogenic mechanism in the development of hypertension (HTN). Oxidative stress, an imbalance between the production and breakdown of ROS, promotes an inflammation-induced vascular damage and endothelial dysfunction, which consequently results in nitric oxide (NO) deficiency, thereby facilitating vasoconstriction leading to increased blood pressure [1]. Oxidative stress occurs primarily through the inhibition of NO bioavailability, via a direct chemical reaction of superoxide anion (O_2_^−^) with NO, resulting in the formation of peroxynitrite. Formation of peroxynitrite may result in further impairment of NO levels and enhance oxidative stress by inhibiting endothelial nitric oxide synthase (eNOS) activity through oxidation of 4-tetrahydrobiopterin (BH_4_), a co-factor of eNOS. This leads to eNOS uncoupling, where eNOS produces superoxide radical instead of NO thereby resulting in increased vasoconstriction, hence pathogenesis of essential HTN [2]. It has been reported from animal studies that the chronic inhibition of NO using *Nω*-nitro-L-arginine methyl ester (L-NAME) induces hypertension through all of the above listed mechanisms [3,4,5]. Our particular interest was the role of L-NAME in increasing ROS as one mechanism of inducing hypertension and how this can be a therapeutic target. Studies have shown that chronic L-NAME administration increases renin and angiotensin converting enzyme (ACE) expression with consequent elevated levels and activity of angiotensin II (Ang II) [6]. Angiotensin II is a potent vasoconstrictor that also has mitogenic, proinflammatory, and profibrotic effects through a variety of signaling pathways, many of which involve ROS. This is primarily through activation of the NADPH oxidase (NOX), which is a major source of ROS in vascular cells [7,8]. It is on this backdrop that a L-NAME induced hypertensive rat model was selected for this study.

Globally, more than 80% of communities rely on herbal remedies to treat various human ailments including HTN [9,10]. Interestingly, the World Health Organisation (WHO) has encouraged the use of traditional medicinal plants for the treatment and prevention of diseases [11]. Dandelion (*T. officinale*), a member of the *Asteracea* family, a perennial herb and commonly regarded as a weed, is native to Eurasia and now generally found elsewhere including North and South America, New Zealand and in Sub-Saharan African countries. *T. officinale* is also an edible leafy vegetable, consumed and used to manage different types of ailments including HTN in many rural communities of South Africa, especially in the Eastern Cape. Studies have shown that plants of the *Asteracea* family possess many pharmacological properties [12]. The *T. officinale* root possesses bifidogenic [13], anti-inflammatory [14,15], and antifibrotic activities [16]. Medicinal plants contain secondary metabolites which are responsible for the therapeutic activities of the plants [17]. A study by Schutz et al. (2005) reported different phytochemical components of *T. officinale* [18]. These secondary metabolites work synergistically to perform their beneficial antioxidant role in medicinal plants. Cortes et al. also emphasized that *T. officinale’s* active ingredients are found in both the roots and leaves, with the leaves containing bitter sesquiterpene lactones such as taraxinic acid and triterpenoids such as cycloartenol, while the roots contain phenolic acids and inulin as well as the aforementioned compounds [19]. Despite the reliance of a huge population on *T. officinale* as a food product and a medicinal plant, scientific data on the safety and in vivo translation of antioxidant properties of this plant is still lacking. Therefore, it is necessary to determine the safety profile and antioxidant properties of *T. officinale* using animal models in this study.

## 2. Materials and Methods

### 2.1. Chemicals and Plant Material

Potassium persulfate (99.5%), sodium carbonate (99%), Trolox (98%), 2,2-azinobis (3-ethylbenothiazoline-6-sulfonic acid) diammonium salt (ABTS) (98%), Folin Ciocalteu’s phenol reagent and Gallic acid (98%) were purchased from Sigma-Aldrich, South Africa. Iron (III) chloride 6-hydrate (97%), dimethyl sulfoxide (DMSO) (99.9%), Iron (II) sulfate 7-hydrate and acetic acid (87%); 2,4,6-Tri(2-pyridyl)-s-triazine (TPTZ) (99%), 2,2-diphenyl-1-picrylhydrazyl (DPPH) (96%), hydrochloric acid (35%), ascorbic acid (99%), sodium chloride (99.9%), and ethanol (99%) were purchased from BioRad, South Africa. 

### 2.2. Plant Collection and Extraction of T. officinale

*T. officinale* was collected from its natural habitat around the Walter Sisulu campus in April 2014. The collected plant was taxonomically identified and authenticated by Dr Immelman of the KEI herbarium, Department of Biological and Environmental Sciences Walter Sisulu University (31°36′08.35′ S, 28°45′02.48′ E) and voucher specimen was prepared and deposited for record purposes (voucher specimen no: AO1/13694). The leaves and the roots of *T. officinale* (TOL and TOR, respectively), were washed in water and air-dried. The dried leaves and roots were separated, ground into a powder using pestle and mortar; and weighed in order to determine the yield of the extract. The powdered leaves and roots were extracted in hydroethanol (70% ethanol solvent) and allowed to shake for 72 h on an automated shaker (Gyro-Rocker, STR9, Staffordshire, UK). The mixtures were filtered with a Buchner funnel through the Whatman No. 1 filter paper. Ethanol in the filtrates was recovered using a rotary evaporator (Laborota 4000 efficient, Heldoph, Germany) under reduced pressure at 40 °C. The water was removed in a fan oven (Labcon 2085K, Germany) at 40 °C. The dry extracts were used for the study. 

### 2.3. Experimental Animals

Wistar rats (150 g to 200 g) and Swiss mice (20 g to 30 g) of both sexes were obtained from the South African Vaccine Initiative (Johannesburg). Animals were accommodated in the animal holding facility in the Department of Biological and Environmental Sciences, and allowed a two-week period for acclimatization during which they had food and water *ad libitum*. Experimental animals were fasted overnight, but allowed access to drinking water before the treatment for proper hydration. Ethical approval for the study was obtained from the Walter Sisulu University Research Ethical Committee (Ethical clearance #: DVC (AA & R) DRD/SREC: Reference No: 03).

#### Induction of HTN in Rats

HTN was induced in Wistar rats using L-NAME (40 mg/kg) administered orally daily for four weeks [5]. Blood pressure was measured at the baseline and monitored weekly during the L-NAME treatment period until animals became hypertensive and randomly distributed into different groups for treatment. Rats with blood pressure (BP) ≥180/120 mmHg were considered hypertensive and therefore included in the study. Animals used for this study were divided into six groups of six (6) rats each (*n* = 6).

- Group I—Control normotensive group (0.9% NaCl).
- Group II—Control hypertensive group (L-NAME (40 mg/kg) 0.9% NaCl)
- Group II—TOL-treated group (500 mg/kg), and
- Group III—TOR-treated group (500 mg/kg).

Animals were treated with drug/extract as per the assigned group at the same time daily for 21 days. Blood pressure was measured once a week between 9:00–12:00 on the same day of the week for each treated group throughout the experimental period using a non-invasive blood pressure machine CODA 8 (Kent Scientific Corporation, Torrington, CT 06790, USA).

### 2.4. Acute Toxicity Study

Acute toxicity of *T. officinale* was assessed in mice using the oral route (p.o) using Lorke’s method [20]. The method involves using thirteen (13) animals for a rapid and economic LD_50_ estimation using the oral route. The experimental procedure was divided into two phases. The first phase consisted of three sub-groups with three animals per group for the dose levels of 10, 100 and 1000 mg/kg bw. The second phase involved the use of sub-groups one animal each for the dose levels of 1000, 1600, 2900 and 5000 mg/kg bw, respectively. Immediately after treatment, each mouse was placed inside a Plexiglas cage and observed for immediate effects during the first 30 min and thereafter for 24 h after treatment for lethal effects. Animals were monitored for additional 14 days to ascertain the delayed effects of *T. officinale*. The LD_50_ of *T. officinale* was estimated as the geometric mean of the lowest dose causing death and the highest dose causing no death according to the formula below.

$${\mathrm{LD}}_{50}\;=\;\surd \left(A\times B\right)$$

where *A* is the maximum dose producing 0% death and *B* is the dose that produces 100% death [20]. From the results of LD_50_, the working dose was determined according to the equation below:

Working Dose ≤1/2 (LD_50_). The working dose of 500 mg/kg for TOL and TOR was selected from a titration curve (data not shown) and therefore, was used in the present in vivo study.

### 2.5. In Vitro Antioxidant Activity of TOL and TOR

The antioxidant activity was assayed to determine the total phenolic and flavonoid contents of TOL/TOR extracts; and characterized for free radical scavenging activity and total antioxidant capacity. All assays were run in triplicate.

#### 2.5.1. Determination of Total Polyphenolic Content (TPC)

A modified method [21] was used for the determination of the total polyphenolic content (TPC). Each extract was mixed with Folin-Ciocalteu reagent (10 time dilution) and 7.5% Na_2_CO_3_ (*w*/*v*). The mixture was vortexed and left for 5 min at room temperature. After incubation, the absorbance was measured at 765 nm and room temperature. Gallic acid was employed as a calibration standard and the results were expressed as mg Gallic acid equivalents (mg GAE) per milligram of the extract.

#### 2.5.2. Determination of Total Flavonoid Content of *T. officinale*

The total flavonoids content of *T. officinale* was determined using a spectrophotometric method [22]. The aluminium chloride reagent was prepared by dissolving 13.3 mg of AlCl_3_ and 40 mg of crystalline sodium acetate with 10 mL of distilled water. For the standard, 0.1 g quercetin (100 mg) was dissolved in a few mL of DMSO, and made up with 100 mL ethanol, to concentrations of 6.25, 12.5, 25, 50 and 100 μg/mL. Three millilitres (3 mL) of sample or standard (Quercetin) were mixed with 1 mL of aluminium chloride (AlCl_3_) reagent. The sample/standard was run in duplicate. The mixture was incubated for 20 min. Absorbance of the colored flavonoid–aluminum complex was measured immediately at a wavelength of 430 nm versus blank. A calibration curve was drawn. Total flavonoid content of the *T. officinale* was expressed as mg quercetin equivalent/mg of the extract. 

#### 2.5.3. Free Radical Scavenging Activity of *T. officinale*

The free radical scavenging activity was described as having activity against the stable form of the synthetic product DPPH (2,2-diphenyl-1-picrylhydrazyl) by the method of Boly et al. [23]. Three millilitres (3 mL) of DPPH in methanol (0.1 mmol/L) was mixed with 1 mL of the crude extract of *T. officinale* in various concentrations (ranging from 0.0078 to 0.5 mg/mL). The mixture was mixed using a Vortex shaker and left to stand in the dark for 30 min at room temperature. Absorbance was read at 517 nm using a UV-spectrophotometer (Phoenix-2000V UV-VIS, Biotech Engineering Management Co. Ltd. (UK), Nicosia, Cyprus). Percentage inhibition was calculated by using the equation:

(MA_b_ − MA_s_/MA_b_) × 100

where MA_b_ = Mean absorbance of the Blank. MA_s_ = the Mean absorbance of the sample. The 50% inhibition capacity of the *T. officinale* equivalent of ascorbic acid was calculated by extrapolating the value from the most linear part of *the T. officinale* percentage inhibition graph.

#### 2.5.4. ABTS^++^ Radical Scavenging Assay

The radical scavenging activity of *T. officinale* was estimated according to a previously reported procedure with slight modification [24]. This is a simple assay which is based on the ability of antioxidants to scavenge ABTS radicals. ABTS^++^ was produced by reacting 7.4 mM of ABTS solution with 2.6 mM potassium persulphate solution, (pH 7.4, in ratio 1:1), and the mixture was kept at room temperature for 12–16 h. The blue-green ABTS working solution was freshly prepared and diluted with methanol to an absorbance of 1.1 at 734 nm. Then, 150 μL of Trolox standard or sample were vigorously mixed with 2850 μL of the ABTS solution and incubated for 1 h at room temperature. The decrease of absorbance was monitored spectrophotometrically at 734 nm (UV-spectrophotometer (Phoenix-2000V UV-VIS, Biotech Engineering Management Co. Ltd. (UK)). The TEAC value was defined as the concentration of Trolox having an equivalent antioxidant activity expressed as µM *TE per milligram weight of the extract (µM TE/mg of extract).*

#### 2.5.5. Determination of Total Antioxidant Capacity (FRAP) of TOL and TOR

The total antioxidant capacity of extracts was determined using the ferric reducing antioxidant power (FRAP) and trolox equivalent antioxidant capacity (TEAC) assay. The FRAP assay quantifies hydrophilic antioxidants as reducing agents in a redox-linked colorimetric method. In the FRAP assay, ferric-tripyridyltriazine is reduced to the ferrous form which has a blue color at low pH. The intensity of the blue color is directly proportional to the reducing power of electron-donating antioxidants present in the test samples [25]. The antioxidant capacity of extracts equivalent of the ascorbic acid was calculated by extrapolating the value from the most linear part of the ascorbic acid standard curve, and expressed as the mg ascorbic acid equivalent/mg of the extract.

### 2.6. In Vivo Antioxidant Activity of TOL and TOR

The in vivo antioxidant activity was assayed to determine the total antioxidant capacity of the extracts that translated into the tissues. All assays were run in triplicate.

#### 2.6.1. Tissue Organ Harvesting and Homogenization

At the end of 21 days of treatment, animals were anaesthetized under CO_2_ inhalation. The heart, liver, kidneys and brain were harvested, cleaned of adhering tissue, weighed and homogenized in a cold 0.01 M phosphate buffer (PBS; pH 7.4) at a ratio of 1:10 (tissue weight: PBS volume); using Potter-Elvehjem homogenizer (Sigma, UK). The homogenate was centrifuged at 4 °C and the supernatant was used for TAC and lipid peroxidation assays.

#### 2.6.2. Determination of Total Antioxidant Capacity (FRAP) in Tissue Homogenate

The ferric reducing antioxidant power (FRAP) assay was carried out according to the procedure of Benzie and Strain (1996) with slight modification [25]. The collected tissues were homogenized using the Potter-Elvehjem homogenizer (Sigma, UK), and the supernatant was used for the assay. The FRAP assay uses antioxidants as reducing agents in a redox-linked colorimetric method. Briefly, the FRAP reagent was prepared from 300 mM acetate buffer (pH 3.6), 10 mM TPTZ solution in 50 mM HCl, and 20 mM iron (III) chloride solution in proportions of 10:1:1 (v/v), respectively. The FRAP reagent was prepared fresh at the time of use at room temperature. Three millilitres (3 mL) of the FRAP reagent was added to 100 μL of the extract and tissue homogenates in different pre-labelled test tubes. The mixture was incubated in a water bath (Labcon, Bergstraße, Germany) at 37 °C for 4 min and the absorbance was read at 593 nm using a UV-spectrophotometer (Phoenix-2000V UV-VIS, Biotech Engineering Management Co. Ltd. (UK)). The ascorbic acid was used to obtain a calibration curve (ranging from 0.78 μg/μL to 100 μg/μL) and the results were expressed as ascorbic acid equivalents in microgram per milligram (μg AAE/mg) of the extract in tissue homogenates.

#### 2.6.3. Lipid Peroxidation Assay in Tissue Homogenate

The lipid peroxidation assay was performed using the thiobarbituric acid reactive substances (TBARs) protocol as described by Oliveira et al. [26]. This assay is based on the reaction of a chromogenic reagent, 2-thiobarbituric acid (TBA), with malondialdehyde (MDA) at room temperature. One molecule of MDA reacts with two molecules of TBA via condensation to yield a chromophore with an absorbance maximum at 532 nm. The collected tissues were homogenized using the Potter-Elvehjem homogenizer (Sigma, UK), and the supernatant was used for the assay. the Thiobarbituric acid-Trichloroacetic acid (TBA-TCA) mixture was prepared by dissolving 0.392 g TBA in 75 mL of 0.25 M HCl, followed by the addition of 5 g TCA, and made up to 100 mL of 0.25 M HCl. In this assay, 500 μL of the TBA-TCA mixture was added to a mixture containing 250 μL of homogenate and 200 μL PBS buffer, in a clean test tube. These test tubes were covered with aluminium foil, and boiled in a water bath at 100 °C for 30 min. The mixture was allowed to cool at room temperature, centrifuged at 2000 rpm for 10 min and the supernatant were transferred into a 96-microwell plate. Absorbance was read on a microplate reader (Biorad, Johannesburg, South Africa) at 540 nm. MDA, a measure of lipid peroxidation was calculated using a molar extinction coefficient of 1.56 × 10^5^ M^−1^ cm^−1^ in the equation

Malondialdehyde concentration (μM) = Absorbance × (540 nm/0.156)

### 2.7. Statistical Analysis

Data were presented as the mean ±Standard error of means (SEM). GraphPad Prism (version 5) was used for statistical analysis of all data. *T*-test was used to compare the in vitro antioxidant activities of TOL and TOR extracts. For all other data, the one-way analysis of variance (ANOVA) followed by Dunnet’s post hoc test was used to compare the means of one group with every other group. The correlation coefficient was calculated using Microsoft office Excel 2010. Statistical significance was set at 5% thus *p* value ≤ 0.05 was considered significant.

## 3. Results

### 3.1. In Vitro Antioxidant Studies

Table 1 shows the total phenolic content, total flavonoid content, free radical scavenging activity, total antioxidant capacity and correlation coefficients of *T. officinale*. The TOL extract showed higher antioxidant potential compared to the TOR extract for all parameters measured. TOL possessed a significantly (*p* < 0.01) higher polyphenol (4.35 ± 0.15 compared to 1.14 ± 0.01 GAE/mg extract) and flavonoid (23.17 ± 0.14 compared to 0.03 ± 0.05 QE/mg extract); DPPH (EC_50_ 0.37 compared to 1.34 mg/mL) and; ABTS (407.5 ± 0.14 compared to 171.5 ± 1.01 TEAC/mg extract) and FRAP (156 ± 5.28 compared to 40 ± 0.3 AAE/mg extract) than TOR. There was a positive correlation between the total antioxidant capacity of TO extracts which was significant (*p* < 0.05) for TOL but not for TOR.

### 3.2. Acute Toxicity Study

In the first phase, doses up to 1000 mg/kg bw did not cause mortality when administered via the oral route. Likewise, no mortality was observed when animals received 1600, 2900 and 5000 mg/kg bw. Moreover, there was no notable changes in animal behaviour and physical appearance 30 min after oral administration of extracts and up to 14 days later. Therefore, the LD_50_ was estimated to be ≥5000 mg/kg bw, orally (p.o) (Table 2).

Table 3 shows the effect of treatment with TOL and TOR on tissue TAC and MDA levels after 21 days. There was a general reduction in the TAC of animals treated with *N*_ω_-Nitro-L-arginine methyl ester (L-Name) (40 mg/kg bw) especially in the heart and liver. Administration of TOL (500 mg/kg bw) improved the TAC in the heart although the response was similar to the control. TOL and TOR (500 mg/kg bw) significantly (*p* < 0.05–0.001) increased TAC in the kidney and brain. Moreover, animals treated with L-Name (40 mg/kg) showed an increase in MDA levels in the treated organs. Treatment with TOL and TOR (500 mg/kg) did not significantly affect MDA levels in the treated organs (Table 3).

## 4. Discussion

The current study investigated the in vitro and in vivo antioxidant properties of *T. officinale*. Results showed that the antioxidant content and effects of TOL were superior to those of TOR and both extracts showed no toxicity (LD_50_ > 5000 mg/kg bw). Antioxidants act as radical scavengers, enzyme inhibitors, metal chelators, hydrogen donors, and singlet oxygen quenchers [27]. Hence, the in vitro antioxidant results obtained in this study suggests a high potential of *T. officinale* to inhibit the actions of free radicals which contributes to the oxidative stress involved in the development of various diseases including HTN.

Acute toxicity studies give clues on the range of doses that can be toxic to animals and could also be used to estimate the therapeutic index of drugs and xenobiotics [28]. LD_50_ is defined as the dose of a substance that is lethal for 50% of the animals in a dose group. Results in the present study showed that LD_50_ of TOL and TOR was greater than 5000 mg/kg (LD_50_ > 5000 mg/kg). Therefore, proposing that TOL and TOR may be regarded as safe. This study corroborates the results presented by Yarnel and Abascal (2009) who showed that up to 6 g/kg, the *T. officinale* extract was not toxic in rabbits [29]. Plant extracts and pharmacological substances with LD_50_ less than 5 mg/kg bw are classified in the range of highly toxic, those with LD_50_ between 5 mg/kg and 5000 mg/kg bw are classified as moderately toxic; while those with LD_50_ more than 5000 mg/kg bw are not toxic [30].

TAC and MDA concentrations are markers of an altered oxidative status caused by NO deficiency. Nitric oxide bioavailability plays a key role in the pathogenesis of oxidative stress-related disease. In addition, the inhibition of NO have been shown to cause elevated ROS, resulting in elevated blood pressure. The current study showed that L-Name treatments generally reduced TAC, and increased lipid peroxidation in all studied tissues except the brain compared to the controls. However, despite the lower in vitro antioxidant capacity (and lack of correlation with polyphenol content) of the root compared to the leaf, the root was equally effective in increasing TAC in the kidney and brain and was more effective in the liver. More so, despite the increased TAC, MDA seems to remain high after treatment with extracts. This may be due to the short duration of treatment. It might be important to have measured the expression of enzymatic antioxidants such as superoxide dismutase (SOD), catalase, glutathione reductase and peroxidase. Expression of heme oxygenases –1 (HO-1) and nuclear factor erythroid 2-like (Nrf2) signalling pathway proteins are used to investigate early antioxidant stimulation at the molecular level [31,32]. In addition to inducing endothelial dysfunction and HTN, many studies report a related elevation of oxidative stress after chronic treatment with L-NAME [33,34,35,36]. Furthermore, hypertension-induced tissue damage is associated with increased oxidative stress [37]. Other studies have reported comparisons of in vitro antioxidant capacity (root and leaf) with general agreement of higher antioxidant capacity in leaf over root [38,39,40,41]. Jamuna et al. reported significant antioxidant activity of both leaf and root extracts [38]. Interestingly, our findings showed that TOL exhibited the highest total phenolic and flavonoid contents than TOR; which was also seen in their free radical scavenging activity and antioxidant capacity (EC_50_ of 0.4 mg/mL and 1.3 mg/mL for TOL and TOR, respectively) performed better in comparison with Baral et al., which shows a direct correlation between TPC and the antioxidant activity of *Ardina cordifolia*; with EC_5.0_ of 48.4 μg/mL and 63.4 μg/mL of the leaf and root extract, respectively [39].

The mechanism responsible for the increase in BP in the L-Name treatment is associated with the NO deficiency [42,43]. Secondary metabolites such as phenolic compounds, saponins, and flavonoids have been shown to exert beneficial effects on the vascular system by their antioxidant properties and lowering blood pressure [44,45]. Our results have demonstrated that TOL possessed a significantly higher polyphenol and flavonoid free radical scavenging activity and; ABTS and TAC than TOR, with a positive correlation between the total antioxidant contents of TO extracts and the antioxidant assay outcomes (R^2^ = 0.93). Polyphenols act on vascular system to regulate the levels and activity of nitric oxide synthase, and therefore the NO bioavailability to the endothelium [46]. This activity involves the ability of polyphenols to interact with kinase signalling pathways and intracellular [Ca^2+^] on eNOS phosphorylation and subsequent NO production [47]. Polyphenols have also been reported to inhibit endothelin-1 (a vasoconstrictor) and endothelial NADPH oxidase (a ROS precursor); and in the long term, angiogenesis and matrix metalloproteinase which are involved in the development of cardiovascular disorders [14]. Flavonoids suppresses NADPH oxidase via the regulation of mitogen-activated protein kinases (MAPK) signalling through receptors for advanced glycation end-products (RAGE), and down regulation of transcription factors such as NF-kB, therefore preventing vascular injury [48,49]. 

There is a paucity of data relating in vitro and in vivo antioxidant activities. Hence, this study is also unique since the in vitro antioxidant activity translates into in vivo activity for the leaf but unexpected for the root which displayed a low in vitro antioxidant activity yet was equally efficacious in vivo. Furthermore, our study is relevant by showing the potency of the leaf part, as opposed to its current traditional claim. 

## 5. Conclusions

Based on the findings in this study, we concluded that TOL possessed better in vitro antioxidant properties than TOR owing to its higher phenolic and flavonoid content; better radical scavenging activity and antioxidant capacity; and that the observed antioxidant property translated into the tissue antioxidant status. 

## Figures and Tables

**Table 1:** In vitro antioxidant studies.

Parameters	TOL	TOR	*p*-Value
ABTS (TEAC/mg extract)	405 ± 0.1	171.5 ± 1.0	*p* < 0.01
DPPH (EC_50_ expressed in mg/mL)	0.4	1.3	*p* < 0.01
TOTAL PHENOLICS (GAE/mg extract)	4.4 ± 0.1	1.1 ± 0.01	*p* < 0.01
TOTAL FLAVONOID (QE/mg extract)	23.2 ± 0.1	0.02 ± 0.1	*p* < 0.01
FRAP (AAE/mg extract)	156.1 ± 5.3	40.4 ± 0.3	*p* < 0.01
Total Phenolics vs FRAP correlation (R^2^)	0.93 *	0.38	*p* < 0.05

Total phenolic and flavonoids content, free radical scavenging activity and antioxidant capacities of TOL and TOR extracts. TOL = *T. officinale* leaf; TOR = *T. officinale* root. TP = Total phenolic content (Gallic acid equivalent/mg extract); TF = Total flavonoid content (Quercetin equivalent/mg extract); EC_50_ = 50% effective concentration; FRAP = Ferric reducing antioxidant power (Ascorbic acid equivalent/mg extract). R^2^ = correlation coefficient. Data are expressed as the mean ± standard error of mean. * *p* < 0.05 compared to TOR.

**Table 2:** Acute toxicity study of *T. officinale* in mice.

Dose mg/kg	Death Patterns after 24 h
	Phase 1
10	0/3
100	0/3
1000	0/3
	Phase 2
1600	0/1
2900	0/1
5000	0/1
LD_50_	≥5000 mg/kg bw, p.o.

**Table 3:** Effect of treatment with TOL and TOR on tissue TAC and MDA levels after 21 days.

Group/Organ	Heart		Liver		Kidney		Brain	
	TAC (mg AAE/mL)	MDA (µM)	TAC (mg AAE/mL)	MDA (µM)	TAC (mg AAE/mL)	MDA (µM)	TAC (mg AAE/mL)	MDA (µM))
Control Norm.	84.9 ± 27.1	1.2 ± 0.1	80.1 ± 2.5	1.1 ± 0.1	77.7 ± 5.5	1.7 ± 0.1	31.1 ± 5.8	1.7 ± 0.1
Control HTN	44.9 ± 0.1	3.2 ± 0.5 ***	36.6 ± 24.1	3.1 ± 0.2 ***	75.1 ± 8.8	3.1 ± 0.4	5.1 ± 1.8 ***	2.6 ± 0.5
TOL	59.3 ± 6.2	1.9 ± 0.1 ^#^	104.1 ± 20.9	3 ± 0.2 ***	312.8 ± 38.1 ***^###^	6.4 ± 0.8 ^###^	15.7 ± 2.2 ***^##^	3 ± 0.4
TOR	43.8 ± 3.5	2.9 ± 0.3 ^##^	186.8 ± 19.4 *^##^	3 ± 0.2 ***	333.3 ± 14.8 ***^###^	4.4 ± 0.3 **	13.7 ± 1.4 ***^#^	2.1 ± 0.3

Control Norm. = Normotensive controls, Control HTN = Hypertensive control; TOL = *T. officinale* leaf extract-treated animals; TOR = *T. officinale* root extract-treated animals. AAE = Ascorbic acid equivalent. Data are presented as mean ±SEM. * *p* < 0.05, ** *p* < 0.01, *** *p* < 0.001 compared to normotensive control animals. ^#^ *p* < 0.05, ^##^ *p* < 0.01, ^###^ *p* < 0.001 compared to hypertensive control animals.
